# A Study of Care Workers’ Wages and Relevant Factors in South Korea

**DOI:** 10.3390/healthcare8020178

**Published:** 2020-06-19

**Authors:** Yun-Young Kim, Seung-Ku Kyoung, Yong-Gab Lee

**Affiliations:** 1 Department of Social Welfare, Jeonbuk National University, Jeonju 54896, Korea; yun2050@jbnu.ac.kr; 2 National Health Insurance Service, 199 Hyeoksin-ro, Wonju-si, Gangwon-do 26464, Korea; hoffnung@nhis.or.kr

**Keywords:** care workers, long-term care, old age and social care, Korean NHIS DB

## Abstract

The purpose of this study is to analyze the wages of South Korea’s care workers as reported by the Korean national health insurance services database. We also try to identify the factors determining the wages of these 1,221,085 care workers. According to the results of the analysis, first, the wage level of care workers is fundamentally low compared to other jobs; second, the labor conditions of home care facilities (compared to those in residential care facilities) are poor, because these depend on wages, which in turn depend on the external environment, such as care institutes’ management; and third, it was verified that the locations of care institutes affect the wages of care workers. As the South Korean population continues to age, the supply of high-quality care workers has important clinical implications for improving the quality of care received by the elderly. Throughout this study, it is argued that the establishment of a social service corporation has become desirable in terms of strengthening public care institutes in Korea.

## 1. Introduction

The Moon Jae-in government is currently discussing the establishment of the social service corporation (or agency). With the growth of social services centered on the private sector in Korea, the demand for “socialization of care” has increased due to limitations in service quality. There are many reasons for including this, but the poor working environment of long-term care workers is considered to be one of the main reasons. The purpose of this study is to analyze the wages of South Korea’s care workers, identify the factors determining these wages and discuss what policy implications can be obtained.

Long-term care workers in Europe are mostly middle-aged women in their forties and older and have low work quality due to low wages and job insecurity [1]. OECD countries have been suffering from such a lack of labor force that some call this situation a care worker crisis. The numbers of long-term care workers in comparison with the proportion of the population aged 65-years-old and older in the UK, the Netherlands and Denmark declined from 1.9 percent, 9.7 percent and 5.5 percent, respectively in 2009, to 1.3 percent, 6 percent and 4 percent, respectively in 2015 [1].

Care workers in Korea are also mostly women in their fifties or sixties, who have graduated from high school. Their wages are less than 50 percent of average wage in all industries, and a high percentage of care work roles are irregular employees. Care workers suffer from working hours that surpass legal limits [2]. Approximately 1.29 million care workers were employed in 2015, but a mere 23.5 percent (305,000) were certificate holders. In addition, in a survey of 1565 care workers (10 percent of all workers working at care institutes in 2014) carried out by the national health insurance corporation, it was found that more than 90 percent of hired care workers are women [3,4].

In Korea’s long-term care insurance policy, the roles of insurer and administrator are taken by the public sector and the role of direct service providers is taken by the private sector. Care workers belonging to old age social care services and approved facilities under the long-term care insurance introduced in 2008 are personnel providing services based on the national certification system. Thus, care worker salaries are determined by respective long-term care institutes’ management within the minimum wage policy. In this situation, long-term care institutes, which must generate profits, may choose to reduce personnel expenses. According to a recent survey, the highest portion of care workers (36.9 percent) chose to change jobs due to low salaries [5]. In the light of this information, South Korean President Moon Jae-in pledged to improve the situation of care workers. However, this is difficult to accomplish since existing private long-term care institutes are fiercely opposed to change.

Long-term care insurance for the elderly provides high-quality services that allow elderly clients to maintain their quality of life. However, as mentioned above, in Korea, care work can be considered unappealing, due to low salaries and unstable employment. This study focuses on wages and begins by exploring current wage levels and the factors affecting them, using the Korean national health insurance services database (Korean NHIS DB).

### 1.1. Understanding Care Work as a Decent Job

Studies of caring labor began in the 1980s when the increase in demand and the problem of supply came to the fore. First and foremost, the 1994 study by Bussemaker and Kersbergen identified that from the perspective of women’s studies, female complementary caring labor is a significant factor that excludes social citizenship, including women’s pensions [6]. Moreover, research on caring labor recognized the sector as in crisis and noted the necessity of reorganizing the welfare state and emerging social policy [7]. In Korea, when the economic crisis occurred in 1997, a crisis of care occurred alongside employment instability for male earners, the participation of housewives in economic activity and the rapid aging of the population. Studies identifying the philosophical meaning of caring shed new light on the overall value of caring and on the position of social policy, emphasizing that the existing welfare state management paradigm should be reshuffled based on caring [8]. Research on the commodification of caring labor, the policy tasks for caring laborer’s working states and improvements in working conditions were also carried out [9]. In particular, after the introduction of elderly long-term care insurance in July 2008, studies regarding the working situation of caring activities and care workers and improvements in working environment and job satisfaction began [10].

Experts in the European Union (EU) assembled professionals from the UN Statistical Commission, the UN Economic Commission for Europe (UNECE), the European Commission (EC) and the International Labor Organization (ILO) to create the task force on the measurement of quality of work. These experts discussed the paradigms of the EC’s ‘quality of work’, the ILO’s ‘decent work’, and the EF’s ’quality of work and employment’, which are used to monitor Europe’s labor conditions. In addition, the taskforce attempted to establish guidelines regarding the international quality of work or recommendations on the measurement of qualitative factors, which can be utilized in both developing and developed countries [11]. In 2008, the taskforce selected seven indicators that can statistically measure aspects of employment including Income and ethics of employment. This represents ILO’s interest in—and efforts to create—‘Care work and care jobs for the future of decent work’.

### 1.2. The Study of Care Workers

With regard to the second indicator, this study focuses on care workers’ wage. Fujisawa and Colombo (2009) [12] reported that the wage level of the low-skilled long-term care laborers in most countries is slightly higher than that of ordinary low-skilled workers. For example, the middle-ranking hourly wage of personnel in the adult care service in the UK is £6.56, which is 14 percent higher than the national minimum wage [13]. In the US, since it is not the norm for residential and home care workers to receive an annual wage hike, and these groups are not protected by the fair labor standards act, the state is excluded from complying with regulations on minimum wage and overtime. According to data published in Germany, the US and New Zealand, nurses working in the long-term care industry work fewer hours and receive lower wages compared to those working in the health industry. Wages have been shown to differ according to region; for example, similar workers in suburban areas of Canada had lower wages. In Korea, the hourly wage of service providers of the elderly caring comprehensive business has been found to be 7425 won ($6.75). On the other hand, since the service providers of personal support services for the handicapped and the house and health visit support program are paid 75 percent of the price of services, their hourly wages have been calculated at 6610 won ($6.01) and 7125 won ($6.48), respectively. Furthermore, service providers of the health support project for mothers and newborn babies earn 8375 won per hour ($7.62). The normal hourly wage of care workers in home care facilities is 6938 won ($6.31), as found by Nam et al. (2013) [14]. Fifty-eight percent received 7000 won ($6.37) and 38 percent earned 6500 won ($5.91), as found by Woo et al. (2013) [3].

Previous studies are meaningful in that they assess real parameters (such as wages) via surveys. However, since these studies were conducted individually, the scope of analysis subjects, the separation of home help services and care facilities, and the division of care workers and workers in other fields do not follow a common standard. In addition, it is difficult to compare or connect various study results, because previous studies have different standards, for example in terms of dividing the wages per month or per hour; not including the allowance for improved labor conditions, which started to be paid in 2013; and analyzing features of the working environment such as working hours. Lastly, since previous studies were based on questionnaires, it was difficult to adequately account for national distribution, the characteristics of institutes and the individual characteristics of care workers.

Normally, wages in the Korean labor market are affected by gender, age, marital status, household members, demographic characteristics, education, forms of employment and social security [13]. According to Lee and Lee (2006) [13], who comprehensively analyzed the Korean labor market, age has the biggest influence on wages; the effect of education is relatively small; the wage gap between occupations is big; and the wage gap between men and women is very big. When taking into account that care work is perceived as a low-paid occupation for middle-aged and old women (since the bottom 10% of the income quintile has greater influence than the top 10% when it comes to the effect of gender on wage), it recalls both Sakellariou’s argument that a policy for low-paid female laborers is needed [15] and Buchinsky’s perspective that wages tend to be higher for a low-wage class with more experience [16].

In order to analyze the factors affecting the wage of care workers, it is necessary to separately take into account the ordinary labor market and the employment status of other care workers. As of September 2015, approximately 94 percent of care workers are women and 16 percent of them are in their forties, 49 percent in their fifties and 29 percent in their sixties. Care workers are hired in a labor market where middle-aged and old women provide caring services based on public assets–expenses regarding the supply of insurance benefits within the frame of long-term care insurance, which is social insurance [17,18]. As a result, it is different from the ordinary labor market and it is necessary to discuss the factors influencing the wages of care workers, for whom a standard wage payment has not been stated (unlike the providers of other caring services). To achieve this, it is necessary to analyze the factors influencing the wages of care workers that take into consideration the characteristics of individuals, regions and care institutes, using data from long-term care institutes.

## 2. Methodology

### 2.1. Analysis of Data

**Figure 1:**
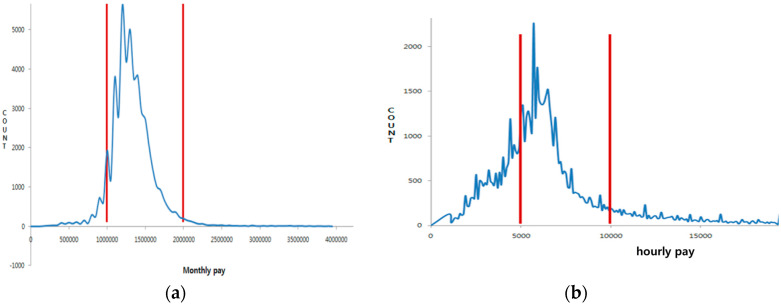
Wage distribution of workers in residential care and home care facilities. (**a**) Distribution of residential care facilities (monthly wage); (**b**) distribution of home care facilities (hourly wage).

The data used by this study to analyze the wages of care workers are from the national health insurance corporation database. The following is the process of data extraction. In the first stage, the sample group of care workers was gathered. The sample subjects are 1,221,085 care workers, from among 1,488,998 care workers certified during the period from 1 July 2008 when elderly long-term care insurance was introduced, to early 2015. In the second stage, to analyze care workers who were working in early 2015, 567,712 were sorted by narrowing down to the office workers in national health insurance. In the third stage, the analysis targets were narrowed down to home help services care workers, who were the biggest proportion (88,443) and the residential care facilities’ care workers, who were working within a standardized system (62,335). In the fourth stage, targets were limited to the care workers at home care facilities (The 2014 national health insurance corporation care worker statistics annual report, 216,358); monthly working hours were restricted to 40–160 h; and extremes were eliminated by limiting the monthly wage of residential care facilities to between 1 and 2 million won ($909 and 1819) and the hourly wage of home help services from 5000 won ($4.55) to 10,000 won ($9.10) (see Figure 1). Based on this standard, the chosen subjects were 49,204 people from residential care facilities and 87,137 from home care facilities (see Table 1).

### 2.2. Analysis Method

Through comparative analysis with existing reports or studies, this study identified differences in terms of wage, age and the characteristics of relevant institutes by implementing descriptive statistical analysis. Moreover, so as to identify the factors affecting the wage of care workers, multiple regression analysis was conducted by having two categories: care workers from residential care facilities and those from home care facilities. The analysis was implemented based on STATA 14.0 (StataCorp LLC, College Station, TX, USA).

### 2.3. Explanation of Variables

According to previous studies, the main factors influencing care workers’ wages in general were age and career. [13,15,16] In addition, the wages of care workers were also influenced by corporate category and regions. [3,13,17,19] The variables were also included. Analytic variables are shown in Table 2. Except for allowance for improvements in labor conditions produced by the national health insurance corporation, the independent variables to analyze as factors influencing the wage of care workers given by care institutes were divided into three categories. The first category of independent variables is ‘the individual characteristics of care workers’, which is comprised of age, monthly working hours, the year in which they acquired their care worker certificate and the number of years they have worked at a care institute. The second category contains two criteria for classifying institutions. One (the foundation of the care institute) is a corporation and a government-operated institution, a facility and a private facility (reference variable), composed of dummy variables. The second (the operation period of the care institute) is a continuous variable. The third category is ’the regional characteristics of care institutes’, which comprises the ratio of care workers to users of the long-term care in the elderly long-term care insurance, the division of city, district and county when it comes to the location of care institute and the division of the location of care institute into capital and noncapital areas.

## 3. Findings

### 3.1. Analysis of Care Workers’ Current Status

In 2017, the average wage for workers in Korea can be seen to be quite low compared to the average wage of 2.9 million won per month [2]. As of early 2015, care workers working at residential care facilities were paid 1.32 million won ($1200) per month, and those at home care facilities received 6573 won ($5.98) per hour. This is similar to the wage levels suggested in previous studies: 1.23 million won ($1120) after tax at residential care facilities found by Jegal (2009) [17]; 1.36 million won ($1240) before tax profits at residential care facilities as found by Cho et al. (2009) [20]; 1.3 million won ($1182) at elderly care facilities by Suh et al. (2012) [21]; monthly wages from 1.2 million won ($1091) to 1.4 million won ($1273) and from 1 million won ($909) to 1.2 million won ($1091) at residential care facilities. Hourly wages of 7000 won ($6.37) (58 percent) and 6500 won ($5.91) (38.2 percent) at home care facilities were found by Woo et al. (2013) [3]; and monthly wages of 1.35 million won ($1236) at residential care facilities and hourly wages of 6938 won ($6.31) were found at home care facilities by Nam et al. (2012) [14] (see Table 3). On the other hand, when the allowance for improvement is included, the monthly wage of care workers in residential care facilities is 1.41 million won ($1290), which is 7.5 percent higher than before and the hourly wage of the care workers working at home care facilities is 7198 won ($6.55), which is 9.5 percent higher.

In the case of Korea, unlike the study by Lee and Lee (2006) [13] that argued that wages normally increase with age, the wages of care workers increase with youth. Table 4 shows the average wage and monthly average working hours based on age. Since there is an absolute shortage of care workers under forty, this age effect can be translated as care institutes giving higher wages to care workers in their forties rather than those in their fifties and sixties.

Table 5 shows the average wage and average monthly working hours based on the date of the foundation of the institute. In the case of the foundation of care institutes, the wages of care workers are ranked in ascending order, as follows: first, local government institutes ($8.42 and $6.49); second, institutes founded by corporate bodies ($8.09 and $6.26); and finally institutes established by individuals ($7.14 and $5.89). In the case of residential care facilities, the proportion of care workers working at care institutes founded by corporate bodies was 50 percent; at care institutes established by local government, 6.5 percent; and the proportion of care workers working at institutes founded by individuals was 44 percent. The treatment of workers at home care facilities is relatively poor and the proportion of the care workers working at care institutes founded by an individual was a massive 74 percent. This supports the claim that the treatment and working environment of home help services are worsening, due to the continuous increase in the number of care institutes established by individuals.

### 3.2. Variables Affecting Wages

The factors affecting the wages of care workers at residential care facilities are presented in Table 6. Regression analysis of the factors affecting the wage of the care workers in residential care facilities showed that all factors had an effect. The wages of care workers who obtained their certificates early proved to be high. The wage levels in institutes founded by corporate bodies and local government were higher than the wage levels in institutes established by individuals. Moreover, wages increased as the operation period of the care institute and workers’ experience increased. Next, when examining regional characteristics, the results were antithetical; while wages in city and district areas are high, the wages of care workers in noncapital areas was higher than the capital area, where most of the care workers reside. This result was derived from the tendency of residential care facilities to be located on the outskirts of big cities. Moreover, the wages of care workers residing in areas with a small elderly population were higher because there are fewer care workers in the area. The two results are equivalent, since the areas with few elderly people are the same as the city areas, and the areas with fewer care workers compared to certifiers is the same as the capital area. Lastly, the study found that it is difficult to take a better job position as a person gets older because the wages of the relatively young generation—those in their forties—were the highest.

The factors affecting the wages of care workers at home care facilities are presented in Table 7. As a result of conducting regression analysis with regard to the factors influencing care workers’ wages at home care facilities, the institutes’ operation period, the city and local area, and the elderly population ratio were different from those of the care workers at residential care facilities. The institutes with short operation periods paid higher wages, and this is the same context as for care workers in the military area (the farming and fishing villages with a high elderly population) and the noncapital area having high wages. Wages increased because the demand for care workers increased. This is related to areas with fewer care workers compared to certifiers, in which there were higher wages. In the case of home care facilities, unlike residential care facilities, they provide care services within farming and fishing communities [22]. However, due to the lack of care workers in these communities, the wages of care workers working at home care facilities are higher than that of the care workers working at home care facilities located in city and district areas.

## 4. Discussion

This study analyzed the wages of care workers by utilizing various database categories in the national health insurance corporation database. To summarize the results, excluding the improvement in the labor conditions fee, the monthly wage of care workers as of early 2015 at residential care facilities was 1.32 million won ($1200)) (1.42 million won ($1291) per year including the improvement of labor conditions fee) and the hourly wage was 6573 won ($5.98) (7198 won ($6.55) including the improvement of labor conditions fee) in home help services. Wages are higher for younger care workers, especially those in their forties; when they are experienced; when the institute is founded by either a corporate body or local government; when the institute has been operating for a long time; and when the institute is located in a noncapital region.

When it comes to factor analysis of the wages paid to care workers at residential care facilities and home care facilities, in the case of those at home care facilities, wages were found to be high when the institute operation term was short; when the region was a military area; and when the region had a large elderly population. Home help services mainly operate in regions of high demand for care protection services and the lack of care workers results in high wages. However, in residential care facilities, since they are mostly located on the outskirts of cities, they are less influenced by regional characteristics than home help services because they are also absorbing the demand of city-dwellers.

When putting together these results, first, the wages of care workers are fundamentally low because their hourly wage is merely 23.2 percent higher than the official minimum wage for those working at home care facilities and 18.6 percent higher for those working at residential care facilities when the improvement of labor conditions fee paid by insurers is not included. Therefore, it is difficult to enhance relatively poor wages with just the hourly 625 won ($0.57) of improvement represented by the labor conditions fee. Second, the scale of care workers working at residential care facilities is similar to those of the institutes established by individuals, corporate bodies and the institutes established by local governments, but the scale of the home help services is overwhelmingly smaller than that of institutes established by individuals. Thus, the status (as expressed in factors such as wages) of care workers working at home care facilities may be lower than that of care workers at residential care facilities. Both the management and supervision of institutes established by individuals and the management and supervision with regard to the overall employment of care workers need to be strengthened. Third, it was verified that the regional characteristics of care institutes have different effects on the wages of the care workers at residential care facilities and home care facilities. In other words, since home help services are affected by regional characteristics more than residential care facilities, suitable approaches for care workers at residential care facilities and home care facilities have to be considered prior to reflecting regional characteristics when developing policy measures regarding their wages.

Therefore, as in the Nordic case, a structural-level policy for the care worker core labor market is needed. According to the results of analysis, it is a reasonable choice to work in a well-run care facility in order to receive high wages as a care worker. In addition, care work at home care facilities appear to be unstable because it depends on the wage level, which in turn depends on the external environment in terms of factors such as the region and the care demand. This is because they are unable to establish their status as professionals, treated according to individual competence. Therefore, it is necessary to strengthen the standardized qualifications system for care workers; to form a compensation system; and to form a ladder structure [10,23] through which to enter the upper labor market.

As states continue to age, the supply of high-quality care workers has important clinical implications for improving the quality of care received by the elderly. Since many bedridden patients are in residential care facilities, care needs to be provided by people who are physically robust (i.e., male and/or young care workers). In the case of home care facilities, it is sometimes required that care workers provide medical services in the private sector without the help of specialists. Hence, high wages are required for high-quality care workers to be recruited and retained.

Strengths and Limitations

This study is meaningful in the context of care worker wage-related studies in the sense that it analyzed a sample of care workers’ wages paid by care institutes, by using the national health insurance DB data. However, since it could not include individual-related variables—education, marriage status, dependent family members and so forth-due to the strengthening of the Personal Information Act, it could not adequately control for demographic characteristics, which have an immense influence on wages. In addition, the data used in this study are not open source. This is a limitation of this study.

## 5. Conclusions

Who is responsible for a compensation system that reflects skilled workers’ wages and job security? Who could implement such a system? Care workers respond to gaps and government policy determines the supply of care workers to the labor market. Guaranteed employment security for care workers ultimately depends on the Korean government [23]. In other words, since care workers are currently employed by private providers, but perform their duties in accordance with the regulations of the Korean government and receive wages via government financing, the relationship between the government and care workers is, in practical terms, a subordinate relationship. In Scandinavian countries with a high number of public care institutions, care work is provided by political decision-making rather than the economic logic of profit-seeking, so that the job of the care worker can be high-quality [5]. In this context, the establishment of a social service corporation has become desirable in terms of strengthening care in Korea.

Throughout the empirical analysis, the objective of improving the labor conditions of care workers has been pursued and is considered a reasonable and worthy aim. In addition, it seems that the private delivery system’s environment is inferior and so the development of social service corporate and community care could be considered, referring to the Nordic model.

## Figures and Tables

**Table 1:** Staff of residential care and home care facilities.

Category	Number of People
Residential care facilities	49,204
Home care facilities	
Part-time care assistants	497
Day and night-care assistants	3345
Visiting home care	43,461
Visiting bath service	39,830
Visiting medical care	3
Support medical devices	1
Total	136,341

**Table 2:** Analytic variables.

Variable		Explanation
Independent Variable	Individual Characteristics	
	Age	Continuous variable
	Year of acquiring certificate	Year 2008 = 2008 Year 2009 = 2009 The years were recorded in order until 2014
	Monthly working hours	Continuous variable (marked in units of time)
	Work experience at an existing care institute	Continuous variable (marked in months)
	Characteristics of care institute	
	Foundation of the care institute	Corporate body Local government foundation institute Institute established by an individual (dummy based on institute established by an individual)
	Operation period of care institute	Continuous variable (marked in months)
	Locations of care institute	
	Number of caregivers in contrast to the number of those who were certified	The number of care workers per certifier of respective cities, districts and counties (separated the residential care facility users and the home care facility users; based on the 2014 Elderly Long-term Care Insurance Statistics Annual Report)
	Ratio of the elderly population	The elderly population ratio of respective cities and counties (based on the 2014 Ministry of Government Administration and Home Affairs Resident Registration Population)
	Separation of location into city and local area	City = 0, Local area = 1
	Separation of location into capital area and noncapital area	Capital area = 1, Noncapital area = 0
Dependent variable	Wage (the improvement of labor conditions not included)	Hourly wage of the caregivers working at home care facilities Monthly wage of the caregivers working at residential care facilities

**Table 3:** Current status of average wage and monthly average working hours.

	Residential Care Facilities		Home Care Facilities	
Average wage 1 ^1^	1.32 million won ($1200) per month	9104 won ($8.28) per hour	620,000 won ($564) per month	6573 won ($5.98) per hour
Average wage 2 ^2^	1.41 million won ($1290) per month	8479 won ($7.71) per hour	680,000 won ($618) per month	7198 won ($6.55) per hour
Monthly average working hours	157.9 hours per month		94.6 hours per month	

^1^ improvement of labor conditions fee is not included. ^2^ allowance for improvement included.

**Table 4:** Average wage and monthly average working hours based on age.

Category			Number of Personnel	Rate (%)	Average Monthly Wage (Won)	Average Hourly Wage (Won)	Monthly Working (h)
Age	Residential care facilities	40 s	7197	18.4	1.37 million ($1246)	8826 ($8.03)	157.8
		50 s	24,088	61.6	1.33 million ($1208)	8533 ($7.76)	157.9
		60 s	7814	20.0	1.25 million ($1132)	7995 ($7.27)	158
	Home care facilities	40 s	5234	15.6	695,000 ($632)	6645 ($6.05)	96
		50 s	17,970	53.7	681,000 ($619)	6575 ($5.98)	95
		60 s	10,269	30.7	665,000 ($604)	6534 ($5.94)	93.2

**Table 5:** Average wage and monthly average working hours based on foundation of institute.

Category			Number of Personnel	Rate (%)	Average Monthly Wage (Won)	Average Hourly Wage (Won)	Monthly Working (h)
Foundation of institute	Residential care facilities	Individual institute	16,417	42.1	1.25 million ($1136)	7850 ($7.14)	157.2
		Corporate institute	19,975	51.2	1.91 million ($1736)	8897 ($8.09)	158.5
		Local government institute	2617	6.7	1.86 million ($1691)	9254 ($8.42)	158
	Home care facilities	Individual institute	25,740	77.5	659,000 ($599)	6478 ($5.89)	93.1
		Corporate institute	7304	22	741,000 ($674)	6877 ($6.26)	99.2
		Local government institute	180	0.5	869,000 ($790)	7134 ($6.49)	112.5

**Table 6:** Factors affecting the wages of the care workers at residential care facilities.

Variable	Wage (The Improvement of Labor Conditions Fee Not Included)		
	B	t	VIF
Year of certificate issue	−9330.8	−20.9 **	1.1
Institute foundation (individual standard)			
Corporate body dummy	122,058.1	65.1 **	1.5
Local government dummy	163,561.7	48.6 **	1.2
Institute operation period	629.1	17.0 **	1.5
Work experience at current institute	2143.0	60.3 **	1.4
Monthly working hours	−664.9	−10.3 **	1.0
City (= 0) and Local area (= 1)	−7173.1	−2.4 *	2.2
Capital area (= 1) and non-capital area (= 0)	−27,385.6	−12.8 **	1.8
Proportion of the elderly population	−521.9	−2.7 *	2.7
Number of caregivers in contrast to the number of certifiers	−61,664.3	−7.7 **	2.1
Age	−3169.8	−22.2 **	1.1
Invariable number	2.024 × 10^7^	22.5 **	
Explanation power (R2)	39.7		

* *p* < 0.05, ** *p* < 0.001.

**Table 7:** The factors affecting the wage of care workers working at home care facilities.

Variable.	Wage (The Improvement of Labor Conditions Fee Not Included)		
	B	t	VIF
Year of certificate issue	−11.3	−3.0 *	1.1
Institute foundation (individual standard)			
Corporate body dummy	368.5	23.3 **	1.1
Local government dummy	606.5	6.9 **	1.0
Institute operation period	−1.2	−4.0 **	1.2
Work experience at current institute	8.9	23.2 **	1.4
Monthly working hours	−6.2	−28.1 **	1.1
City (= 0) and Local area (= 1)	143.2	4.9 **	2.2
Capital area (= 1) and noncapital area (= 0)	−185.8	−10.2 **	1.8
Proportion of the elderly population	9.2	5.2 **	2.6
Number of caregivers in contrast to the number of certifiers	−357.8	−6.4 **	1.8
Age	−4.5	−4.4 **	1.0
Invariable number	30,106.5	3.9 **	
Explanation power (R2)	24.3%		

* *p* < 0.05, ** *p* < 0.001.
